# Experimental Infection in Mice with *Cryptosporidium* Isolated from Humans

**DOI:** 10.3390/pathogens14090843

**Published:** 2025-08-23

**Authors:** Rodica Georgiana Dărăbuș, Marius Stelian Ilie, Gheorghe Dărăbuș, Sorin Morariu, Diana Maria Dărăbuș, Narcisa Mederle, Mirela Imre, Ioan Ovidiu Sîrbu, Tudor Rareș Olariu

**Affiliations:** 1 Discipline of Parasitology, Department of Infectious Diseases, Victor Babes University of Medicine and Pharmacy, 300041 Timisoara, Romania; georgiana.darabus@umft.ro (R.G.D.); rolariu@umft.ro (T.R.O.); 2 Center for Diagnosis and Study of Parasitic Diseases, Department of Infectious Disease, Victor Babes University of Medicine and Pharmacy, 300041 Timisoara, Romania; 3 Discipline of Parasitology, Faculty of Veterinary Medicine, University Life Sci King Michael I, 300645 Timisoara, Romania; gheorghe.darabus@fmvt.ro (G.D.); sorin.morariu@fmvt.ro (S.M.); narcisamederle@usvt.ro (N.M.); mirela.imre@usvt.ro (M.I.); 4 Department of Ophthalmology, Victor Babes University of Medicine and Pharmacy, 300041 Timisoara, Romania; 5 Department of Biochemistry, “Victor Babes” University of Medicine and Pharmacy, 300041 Timisoara, Romania; ovidiu.sirbu@umft.ro; 6 Center for Complex Network Science, “Victor Babes” University of Medicine and Pharmacy, 300041 Timisoara, Romania

**Keywords:** *Cryptosporidium canis*, *C. ryanae*, *C. parvum*, mice, experimental infection

## Abstract

*Cryptosporidium* is a genus of protozoa that infects the gastrointestinal and respiratory epithelium of various host species. The aim of this study was to perform experimental infection in conventional mice with three *Cryptosporidium* species isolated from humans. The three *Cryptosporidium* species, namely *Cryptosporidium canis*, *Cryptosporidium parvum*, and *Cryptosporidium ryanae*, were obtained from fecal samples collected from patients hospitalized in an infectious disease hospital. The mice, from 10-day-old litters kept with their mother, were divided into three groups and orally infected with one of the *Cryptosporidium* species. The first oocysts were identified in the feces of the mice four days post-infection. The infection was successful with all three *Cryptosporidium* species, but the infection level (expressed as the number of oocysts per microscopic field) was low. The infection was detected using a rapid immunochromatographic test 40 days post-infection. Furthermore, starting on the 17th day after infection, the mothers also tested positive on the rapid immunochromatographic test, having been negative until that point. It was concluded that mice could represent a source of infection for the three *Cryptosporidium* species in other susceptible species, including humans. No behavioral changes or diarrhea were observed in any of the experimental cases.

## 1. Introduction

*Cryptosporidium* species are parasitic protozoa localized in the digestive and respiratory epithelium of mammals including humans, birds, reptiles, and fish [1].

In cryptosporidiosis, the clinical course can range from asymptomatic shedding of oocysts to severe clinical manifestations, depending on the host’s immune status. In immunocompetent individuals, the disease is usually self-limiting and can resolve even without treatment [2]. Due to its low host specificity, only a single species, *Cryptosporidium parvum*, was initially recognized [3]. Later, in 2013, Slapeta reviewed molecular and experimental studies conducted worldwide and summarized evidence supporting the recognition of 30 valid *Cryptosporidium* species [4]. Currently, at least 44 valid species and over 120 genotypes are recognized [5].

Among these, nearly 20 *Cryptosporidium* species are zoonotic, with *Cryptosporidium hominis*, *C. parvum*, *C. meleagridis*, *C. canis*, and *C. felis* being the most frequently detected species in humans [5,6,7].

*Cryptosporidium parvum* is the type species for mammals and was first identified in the intestine of the house mouse (*Mus musculus*) by Ernest Edward Tyzzer in North America [8]. It primarily infects the ileum, especially the epithelial domes in Peyer’s patches, but it can also be found in other regions of the intestine. Infection with *C. parvum* has been recorded in a large number of mammalian species, including humans, but is most prevalent in cattle, sheep, and goats [3,9,10,11].

*Cryptosporidium canis* cannot be morphologically distinguished from the oocysts of *C. parvum* and shares common surface antigens with it. While it can infect cattle, it is unable to cause infection in mice [12]. Confirmed infections with *C. canis* have been reported in dogs, coyotes, foxes, and humans [13,14]. While experimental infections with *C. parvum* and *C. meleagridis* have been demonstrated in mice [10,15], infection with *C. canis* was successfully demonstrated in immunosuppressed dogs but could not be achieved in immunocompetent dogs or SCID mice [16].

A study conducted in Italy reported the presence of *Cryptosporidium* spp. on fresh produce, including salads and berries. Molecular analysis identified *Cryptosporidium ryanae*, *C. bovis*, *C. xiaoi*, and *C. ubiquitum* on these food items [17]. Additionally, *C. ryanae* was also detected in water samples. These findings suggest that vegetables, fruits, and water may serve as potential sources of *C. ryanae* infection in humans. In a separate study from 2019, Das et al. [18] identified *C. ryanae* in individuals working on cattle farms. *Cryptosporidium ryanae* has been identified in calves and was reported to be non-infectious for BALB/c mice and lambs [19]. However, this species was later detected in rats (*Rattus norvegicus*) [20].

The literature on interspecies transmission is limited, and recent data are almost nonexistent. This study aimed to determine the potential of rodents in general, and mice in particular, as a source of infection for humans through experimental infections with various *Cryptosporidium* species isolated from humans.

Additionally, this study developed a murine model for experimental infection with multiple *Cryptosporidium* species originating from humans. The study also sought to resolve certain controversies regarding the interspecies transmission of three *Cryptosporidium* species isolated from humans.

## 2. Materials and Methods

### 2.1. Inoculum

**Figure 1:**
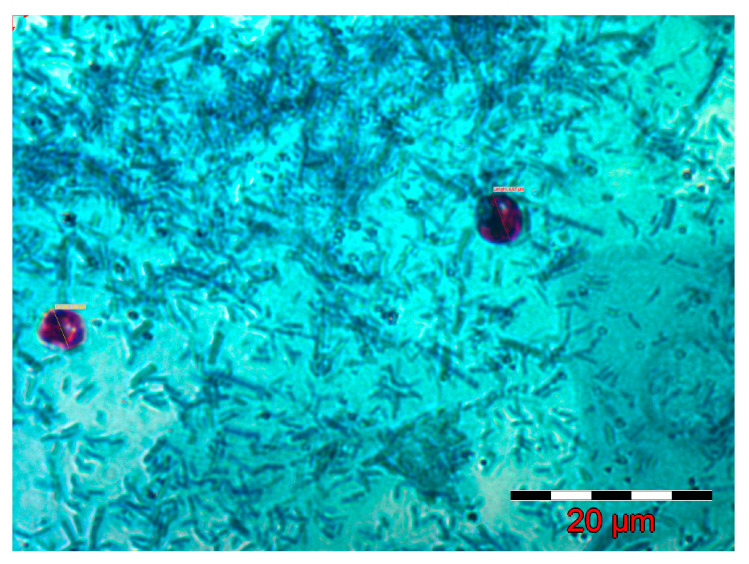
*Cryptosporidium* spp. oocysts in a positive fecal smear (Ziehl-Neelsen method).

The inoculum was prepared from fecal samples obtained from humans infected with *Cryptosporidium*. The positivity of the human fecal samples was determined using the modified Ziehl-Neelsen method described by Henriksen (Figure 1). These samples were subjected to PCR analysis following the technique described by Xiao and Ryan (2008) with slight modifications [21].

The fecal samples in which *C. canis* and *C. parvum* were identified originated from humans who were dog owners, while the samples containing *C. ryanae* came from members of an urban family with no contact with animals. The identification of *C. ryanae* in humans is reported for the first time in Romania (unpublished data). The inoculum was prepared according to the following protocol:

Homogenization of feces in a 1:10 ratio with tap water.

1. Consecutive filtration through sieves with mesh sizes of 500 µm and 100 µm and five layers of gauze.
2. Centrifugation at 1000 rpm followed by two successive washes with phosphate-buffered saline (PBS).
3. Flotation of the sediment in Sheather’s sucrose solution.
4. Recovery of oocysts using a pipette and washing in PBS.
5. Addition of penicillin (100 IU/mL) and streptomycin (1 mg/mL) to the oocyst suspension.

Three inocula were prepared:

- *C. parvum* containing 3350 oocysts/mL,
- *C. ryanae* containing 3650 oocysts/mL,
- *C. canis* containing 3100 oocysts/mL.

The concentration of oocysts was determined using a Bürker-Türk hemocytometer.

### 2.2. Experimental Animals

For the experiment, three pregnant females of conventional white mice (mated on the same day) were used. They were housed in separate cages and provided with a standard rodent diet and water *ad libitum*.

The offspring of these females, aged 10 days, were used for the experimental study. Group I consisted of 9 mice, Group II of 10 mice, and Group III of 11 mice. The groups were orally infected with 0.05 mL of inoculum containing *C. parvum*, *C. ryanae* and *C. canis*, respectively (Table 1).

### 2.3. Assays Performed

On days 3, 4, 7, and 10 post-infection (p.i.), individual fecal samples were collected. Each sample was subjected to a direct coproscopic examination of the feces [9]. For each sample, 10 fields were examined, and the number of oocysts per field was calculated as the average from the 10 fields.

Due to dietary changes (no longer exclusively milk-based), the feces were unsuitable for smear analysis from day 14 p.i. onward, and the individual fecal samples (Table 1) were analyzed using the CerTest Crypto (San Mateo de Gállego Zaragoza, Spain) rapid immunochromatographic test. The CerTest Crypto card test provides a straightforward and highly sensitive screening method for the presumptive diagnosis of cryptosporidiosis [22].

The three adult females were also examined for fecal oocysts (CerTest Crypto test) on days 0, 3, and 4 and post-experimental infection of their pups.

### 2.4. Ethical Approval

This work involved the use of experimental animals and was approved by the Ethics Committee of the Faculty of Veterinary Medicine, Timișoara, under approval number 523/3 November 2025.

## 3. Results

**Figure 2:**
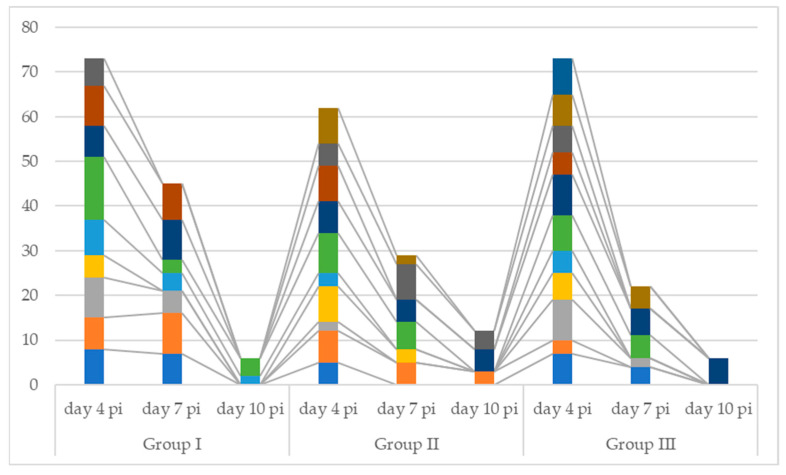
Graphical representation of the dynamics of oocyst elimination in the three groups of mice (p.i.—post infection). Each color in the chart represents the number of oocysts counted for each positive mouse in the respective group.

**Figure 3:**
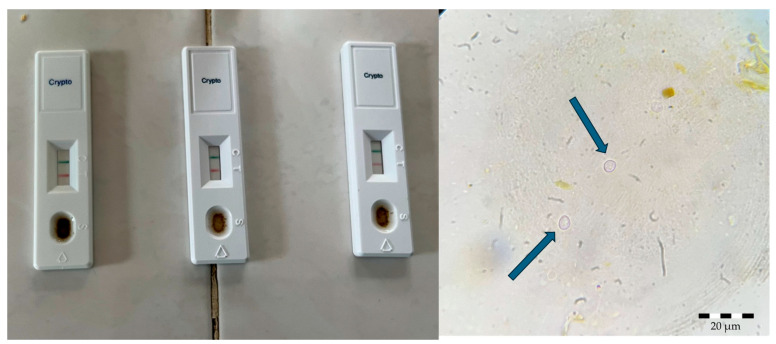
Positive rapid tests (**left**) and native smear of *Cryptosporidium* spp. oocysts (**right**).

The results of the experimental infection with *Cryptosporidium* in mice are presented in Table 1, Table 2 and Table 3 and Figure 2 and Figure 3. At 4 days post-infection (p.i.), all infected mice, regardless of the *Cryptosporidium* species, were shedding oocysts, although in low numbers (<1 oocyst/field), with one exception: a mouse infected with *C. parvum* exhibited a higher number of oocysts.

Gradually, by day 10 p.i., the number of oocysts decreased, likely due to the difficulty in detecting them in native smears, as the oocysts may have been obscured by food residues due to the diet no longer being exclusively milk-based. Subsequently, using rapid immunochromatographic tests, we noted that all experimental mice, regardless of the *Cryptosporidium* species, tested positive for cryptosporidial infection at two weeks post-infection.

Furthermore, on day 17 p.i., *Cryptosporidium* was identified in the mothers of the mice using the rapid test. At 40 days post-infection, both the mice and their mothers tested positive for *Cryptosporidium* infection as determined by the rapid immunochromatographic tests.

No significant changes regarding appetite or fecal consistency were observed. No animals died during the experiment.

## 4. Discussion

While the transmission of *Cryptosporidium* within the same species is widely accepted, interspecies transmission remains a topic of significant debate, with contradictory findings and a lack of full consensus [7,11,16,23,24,25].

Initially, it was believed that the genus *Cryptosporidium* consisted of a single species [3] due to its ease of transmission from calves to several mammalian species. However, current molecular analyses have revealed the existence of a large number of species and strains [5]. Nevertheless, the issue of interspecies transmission remains partially unresolved.

Experimental infections [11] demonstrated that *C. parvum* oocysts isolated from calves were infectious to seven mammalian species (lambs, rabbits, dogs, cats, mice, rats, and guinea pigs) aged 3 to 12 days, but not to 3-day-old chickens. Although infection was 100% successful in mammals, differences were observed in infection intensity, prepatent periods, and patent periods [10].

Our study aimed to determine whether there are differences in *Cryptosporidium* infections in mice with various species isolated from humans. It is known that livestock and carnivores can serve as sources of infection for humans [10,26]. Similarly, rodents can act as reservoirs for a wide range of infectious diseases.

Our experiment also sought to address some controversies regarding the experimental transmission of multiple *Cryptosporidium* species. For instance, Del Coco et al. (2012) successfully infected 3-week-old mice with *C. parvum* subtype IIaA21G1R1 only after immunosuppression with dexamethasone. Similarly, Taha et al. (2023) achieved infection of Swiss Albino laboratory mice with *C. parvum* following immunosuppression with dexamethasone [27,28].

On the other hand, Cui et al. (2018) reported that *C. canis* oocysts are not infectious to immunocompetent dogs or SCID mice. According to these authors, experimental infection with *C. canis* is only successful in dogs immunosuppressed with dexamethasone [16].

It is important to note that *C. ryanae* isolated from calves has been shown to produce oocysts that are not infectious to BALB/c mice or lambs, as demonstrated by Fayer et al. (2008). This suggests a degree of host specificity for *C. ryanae*, potentially limiting its zoonotic potential and cross-species transmission. However, further studies are necessary to fully elucidate the host range and infectivity patterns of this species, particularly in the context of public health [19].

However, *C. ryanae* has also been detected in rats inhabiting dairy farms, as reported by Ježková et al. (2021). This finding suggests that rodents may act as potential reservoirs or mechanical carriers for *C. ryanae* within farm environments, which could contribute to its environmental persistence and dissemination among livestock. Further research is required to clarify the epidemiological role of rodents in the transmission dynamics of this species [20].

According to our results, in experimental infections performed on conventional mice, the infection was successfully established regardless of the *Cryptosporidium* species used. Furthermore, it can be concluded that mice can serve as a source of infection, as demonstrated by both the successful experimental infection in mice and the transmission of infection to the mothers of the mice.

This latter aspect indicates that not only do young mice become infected and serve as a source of infection, but adult mice can also contribute to transmission. The persistence of the infection at 40 days p.i. is likely due to the contaminated environment caused by repeated oocyst shedding, leading to successive reinfections.

In contrast to our findings, Sherwood et al. (1982) [29] achieved only transient infections in mice aged 21 days or older. These differences could be attributed to varying experimental conditions, as the authors used SPP mice rather than conventional mice. However, *Cryptosporidium* infection was successfully established in mice immunosuppressed with dexamethasone in experimental infections [30].

Furthermore, it appears that in mice, *Cryptosporidium* does not cause lesions that result in clinically observable changes in general condition or digestive disturbances. This enhances their role as a source of infection for other mammalian species. Similarly, other authors [29] have also concluded that mice are susceptible to subclinical infections unless they are immunosuppressed [27,31].

## 5. Conclusions

The present study demonstrates that experimental infections with *C. canis*, *C. ryanae*, and *C. parvum* isolates of human origin can be successfully reproduced in conventional mice, regardless of the animals’ age. These findings contribute to clarifying previous inconsistencies regarding the host specificity and interspecies potential transmission of these *Cryptosporidium* species. Moreover, the results emphasize that conventional mice can act as competent hosts and potential sources of *Cryptosporidium* infection for other susceptible animal species, including humans, thereby underlining their possible role in the epidemiological cycle of the parasite.

Additionally, this study reports, for the first time, the identification of *C. ryanae* in humans in Romania, representing a novel contribution to the national and regional epidemiological data. However, the zoonotic potential of *C. ryanae* remains insufficiently understood as no dedicated studies have been conducted to date to assess its ability to infect humans and its potential impact on public health. Therefore, further comprehensive research, including molecular characterization and cross-species infectivity trials, is urgently needed to elucidate the transmission dynamics, host range, and possible zoonotic implications of *C. ryanae*.

Collectively, these findings provide new insights into the host adaptability of *Cryptosporidium* species and highlight the importance of considering non-traditional animal models, such as mice, in experimental studies aimed at better understanding the epidemiology and transmission pathways of cryptosporidiosis.

## Figures and Tables

**Table 1:** Experimental infection of mice with *Cryptosporidium* species isolated from humans.

Day p.i.	Examination Method	GROUP I Infected with *C. parvum* Infected/Total Mice	GROUP II Infected with *C. ryanae* INFECTED/Total Mice	GROUP III Infected with *C. canis* Infected/Total Mice	Mother Mice Infected/Total Mice
3	Direct examination	9/9−	10/10−	11/11−	3/3− *
4	Direct examination	8/9±; 1/9+	10/10±	11/11±	3/3− *
7	Direct examination	7/9±; 2/9−	6/10±; 4/10−	5/11±; 6/11−	ND
10	Direct examination	2/9±; 7/9−	3/10±; 7/10−	1/11±; 10/11−	ND
14	Quick Crypto Test	9/9+	10/10+	11/11+	ND
17	Quick Crypto Test	9/9+	10/10+	11/11+	3/3+
23	Quick Crypto Test	9/9+	10/10+	11/11+	3/3+
31	Quick Crypto Test	9/9+	10/10+	11/11+	3/3+
40	Quick Crypto Test	9/9+	10/10+	11/11+	3/3+

p.i. = post infection; ± = <1 oocyst/field; + = 1–3 oocyst/field; − = negative; + = positive; − * = negative by Quick Crypto Test; ND—not determined.

**Table 2:** The dynamics of oocyst elimination (oocyst per 10 fields) in the three groups of mice that were subjected to the experiment.

No Mice	Group I			Group II			Group III		
	Day 4 p.i.	Day 7 p.i.	Day 10 p.i.	Day 4 p.i.	Day 7 p.i.	Day 10 p.i.	Day 4 p.i.	Day 7 p.i.	Day 10 p.i.
1	8	7	0	5	0	0	7	4	0
2	7	9	0	7	5	3	3	0	0
3	9	5	0	2	0	0	9	2	0
4	5	0	0	8	3	0	6	0	0
5	8	4	2	3	0	0	5	0	0
6	14	3	4	9	6	0	8	5	0
7	7	9	0	7	5	5	9	6	6
8	9	8	0	8	0	0	5	0	0
9	6	0	0	5	8	4	6	0	0
10				8	2	0	7	5	0
11							8	0	0

p.i.—post infection.

**Table 3:** Descriptive statistics of oocyst elimination dynamics in the three groups of mice.

	Group I			Group II			Group III		
	Day 4 p.i.	Day 7 p.i.	Day 10 p.i.	Day 4 p.i.	Day 7 p.i.	Day 10 p.i.	Day 4 p.i.	Day 7 p.i.	Day 10 p.i.
Mean	8.111111	5	0.666667	6.2	2.9	1.2	6.636364	2	0.545455
Standard Error	0.857069	1.178511	0.471405	0.742369	0.936305	0.628932	0.560401	0.750757	0.545455
Median	8	5	0	7	2.5	0	7	0	0
Mode	8	9	0	8	0	0	7	0	0
Standard Deviation	2.571208	3.535534	1.414214	2.347576	2.960856	1.988858	1.858641	2.48998	1.809068
Sample Variance	6.611111	12.5	2	5.511111	8.766667	3.955556	3.454545	6.2	3.272727
Kurtosis	3.521119	−1.35543	4	−0.64606	−1.24007	−0.2244	−0.18943	−1.63892	11
Skewness	1.544483	−0.34911	2.12132	−0.74974	0.442401	1.245704	−0.49083	0.617529	3.316625
Range	9	9	4	7	8	5	6	6	6
Minimum	5	0	0	2	0	0	3	0	0
Maximum	14	9	4	9	8	5	9	6	6
Sum	73	45	6	62	29	12	73	22	6
Count	9	9	9	10	10	10	11	11	11
Confidence Level (95.0%)	1.976406	2.717652	1.087061	1.679354	2.118069	1.422743	1.248652	1.672791	1.215348

p.i.—post infection.

## Data Availability

The original contributions presented in this study are included in the article. Further inquiries can be directed to the corresponding authors.
